# Commercialising everyday distress: neurasthenia and traditional Chinese medicine in colonial Hong Kong, 1950s to 1980s

**DOI:** 10.1017/mdh.2024.7

**Published:** 2024-10

**Authors:** Kelvin Chan

**Affiliations:** Department of History and Classical Studies, McGill University, Montreal, Quebec, Canada

**Keywords:** Neurasthenia, traditional Chinese medicine, colonial Hong Kong, proprietary medicine, social history of medicine, psychiatry

## Abstract

The persistent use of neurasthenia in Asia, an out-dated diagnostic category in modern psychiatry, has confounded many psychiatrists from the 1960s. This paper attempts to understand the prevalence of neurasthenia among the lay public in post-World War II Hong Kong. It examines the social history of psychiatry and focuses on the roles of traditional Chinese medicine in shaping public perceptions and responses towards neurasthenia. This research reveals that, when psychiatrists discarded the term as an ineffective label in the 1950s, practitioners and pharmaceutical companies of Chinese medicine seized on the chance to reinvent themselves as experts in neurasthenia. By commericialising everyday distress, they provided affordable, accessible and culturally familiar healing options to the Chinese public. A case study of neurasthenia, therefore, is not simply about changing disease categories but an important example to illustrate the tensions between traditional medicine and Western psychiatry in Asia.

## Introduction

Neurasthenia occupies a unique place in the history of psychiatry. George M. Beard, an American neurologist, first proposed the term in 1869 to describe several syndromes, including anxiety, fatigue, headaches, insomnia, bodily pain and irritable mood.^1^ Neurasthenia became a common diagnosis for functional nervous diseases in the United States by the late nineteenth century.^2^ What made neurasthenia unusual was its ‘afterlife’ in the mid-twentieth century. It faded into an obsolete disease category in 1940s United States due, in part, to its imprecision.^3^ Yet, the medical label had a lasting impact elsewhere. Tsung-Yi Lin, a Taiwanese psychiatrist and former director of the Division of Mental Health in the World Health Organisation, highlighted the importance of studying neurasthenia because the term was ‘in use by perhaps half of the population living on this globe in a variety of medical and psychosocial contexts’ in the 1970s and 1980s.^4^ Some psychiatrists described the puzzling popularity of neurasthenia as a reflection of the mistrust of traditional medicine. Therefore, to these psychiatrists, the public needed to ‘learn the correct health concepts’ from the experts.^5^ Neurasthenia became the focus of anthropological research since the 1970s. Their fieldwork offered valuable observation about neurasthenia: 1) it was prevalent in Asia as a somatic expression of depression; 2) it was considered by patients as a somatic problem and therefore exempted from stigmatisation and 3) it blended with traditional Chinese medicine in the Chinese-speaking world.^6^

Historians have not yet fully addressed the fundamental question of why and how ‘neurasthenia’ persisted long after it was discarded by mainstream psychiatry. Historians in recent decades have explored the history of neurasthenia in Asia and Africa in the nineteenth and early twentieth century. This scholarship revolves around the framing of neurasthenia as a ‘disease of civilisation’: colonists took the label as a racial marker to distinguish the civilized White population from the primitive local,^7^ or the colonised appropriated the term to signify their level of civilisation and resistance to the colonial norms.^8^ More recently, historians have begun to shift the focus to the mid and late twentieth century. For instance, Wen-Ji Wang illustrates how neurasthenia became a popular idiom of expressing distress in the early twentieth century China, but the People’s Republic of China elevated neurasthenia to be a national problem due to its symptoms of unproductivity and passivity, making it an undesirable illness in a socialist state.^9^ The political influence, however, could not explain why neurasthenia remained popular outside of Communist China, such as Hong Kong, Taiwan and the Chinese migrants in Southeast Asia and North America. Looking beyond the state’s agenda, Howard Chiang examines another mental illness that was closely associated with Chinese culture, koro (縮陽), and shows that psychiatrists outside of continental China conceptualized koro by appropriating traditional Chinese medical theories and culture.^10^ Despite this important work, the scholarly focus is still predominately professionally trained psychiatrists.

This study approaches the question of neurasthenia by centring on the roles of traditional medicine, an understudied aspect assumed by many historians to be marginal to modern psychiatry. Focusing on the social history of neurasthenia, this study suggests practitioners and retailors of traditional Chinese medicine were crucial actors in the care of mind, complementing and sometimes competing with psychiatrists. It was precisely the psychiatrists’ discarding of neurasthenia as a clinical label from the 1950s that turned neurasthenia into a potential territory of traditional healing. Unlike antidepressants that were strictly controlled under colonial legislation, traditional medicine was free from such regulation because the colonial government was reluctant to intervene into Chinese customs. Practitioners and retailers of Chinese medicine seized on the chance to reinvent themselves as experts of neurasthenia, publishing extensively, and promoting antineurasthenic treatments and proprietary medicine. Despite the biomedical profession’s view of Chinese medicine as unscientific and superstitious, many patients accepted Chinese medicine in treating neurasthenia in everyday life due to its cultural familiarity, affordability and accessibility. This study argues that traditional Chinese medicine was essential to maintaining the popularity of neurasthenia. The commercialization of illness and psychological distress, in this sense, explains why neurasthenia persisted outside of continental China. Hong Kong, in particular, serves as a useful comparison to understand similar tensions in Asia, including the strict colonial legislation of antidepressants, the thriving business of traditional medicine and its connections with Chinese migrants. After all, an historical analysis of neurasthenia is not simply about changing disease categories but constitutes an important example to illustrate the tensions between traditional medicine and Western psychiatry in Asia.

This paper first reviews the broader history of neurasthenia in Hong Kong from the nineteenth to the twentieth century. It then focuses on three interconnected perspectives: practitioners of Chinese medicine, retailers of proprietary medicine and patients. It highlights how neurasthenia was interpreted, commercialised and experienced and utilises various materials that have not been fully explored by scholars: medical advertisements, catalogues of proprietary medicine, patients’ narratives in medical columns, popular self-care booklets and guides, medical magazines and government records.

## Neurasthenia in colonial Hong Kong

From the late nineteenth century, Europeans and Americans were concerned about tropical neurasthenia as part of the broader colonial anxiety over White populations settling in the tropics. In modern China, the theories about the causes of White breakdown ranged from lack of sex, dislocation from home and civilisation or even failure to acclimate to Chinese customs and culture.^11^ The typical treatment of tropical neurasthenia was relocation away from the tropics. Hutchison, the superintendent of imports and exports in Hong Kong, who suffered from severe neurasthenia, was advised by his doctor to leave the colony during the summer and to take at least six months of complete rest in a good climate for recovery.^12^ More importantly, being free from neurasthenia and nervous affection was a prerequisite of the recruitment of European police officers.^13^ At the same time, neurasthenia could be an excuse to disqualify officials from employment. One example was Robert Andrew Dermod Forrest, a cadet officer in Hong Kong, the first police magistrate in 1938 and immigration officer in 1940, who suffered from acute nephritis and neurasthenia. In his own account of his medical history, he attributed the cause of his illness to the lasting consequences of the humid climate and the conditions of urban life in Hong Kong. After evaluating his medical records of Forrest, the Colonial Office found him unfit for the post and decided to invalidate his service.^14^

Neurasthenia was a highly racialised illness. Early missionaries and medical journals posited that Chinese, who were free from a high-pressured lifestyle, would rarely become insane and neurasthenic. Although the records of mental asylums in the early twentieth century revealed an increasing number of neurasthenia cases among the Chinese, missionaries did not view this evidence as proof of civilisation. In the spirit of the civilising mission, the missionaries instead attributed Chinese insanity to the backward Chinese customs and traditions, such as opium or daily sexual intercourse.^15^ One commentary in Hong Kong newspapers associated racial immunity from diseases with Chinese backwardness: ‘their towns are guiltless of sanitation, but typhoid finds no footing in China; their canals are drains, but the Chinaman drinks from them and escapes dysentery… and neurasthenia is as uncommon as organic heart disease’.^16^ However, the racial assumptions of the Chinese as a people free from insanity remained ideological. From 1910 to 1939, lunatic asylums in Hong Kong placed neurasthenia under the category of brain or nervous system disease. It treated a total of 460 neurasthenic patients, including both Chinese and European.^17^ To the colonial government, neurasthenia was not a significant cause of mental illness among admitted patients into lunatic asylums and was negligent compared to other more deadly diseases, such as plague. In other words, neurasthenia was highly racialised, but not a threat to governance.

By the mid-twentieth century, neurasthenia quickly became an outdated term among mental health professionals. In the context of the United States, neurasthenia had grown out of fashion. Doctors sought more precise diagnoses and scientific evidence to define the highly popularised disease, whereas pharmaceutical companies responded by tightening regulations for advertising proprietary medicine and thereby shifted away from neurasthenia. More specific terms – nervous breakdown, neurosis and psychosis – became favourable and replaced neurasthenia.^18^ Meanwhile, in Hong Kong, after World War II, psychiatrists also removed neurasthenia as a diagnostic label. In 1948, Pow Meng Yap (葉寶明), trained in Cambridge and later the Maudsley Hospital, took up the position of medical superintendent of the Victoria Mental Hospital and was the first professionally trained psychiatrist in Hong Kong. A modern system of mental health services gradually emerged, and the care of mental health became a professional territory from the 1950s: more mental hospitals and outpatient clinics were established, psychiatric training became part of the curriculum in medical school and government sponsored graduate training at the Maudsley Hospital.^19^ Neurasthenia was removed from the Medical Annual Report in 1950 when Yap restructured and renewed the diagnostic categories used in official reports.^20^

The 1960s was a turning point in the history of psychiatry in Hong Kong. In 1961, the colonial government established the first mental health hospital and since then admitted unprecedented number of patients. In 1962, the Castle Peak Hospital had already admitted 3 495 cases and had to call for additional beds in other wards.^21^ In 1966, Wai Kiu Yat Po commented on the overcrowded situation in Castle Peak and reported that Hong Kong had now become a ‘breeding ground’ of insanity due to social immorality. It even made a seemingly startling estimation that Hong Kong had over 50 000 mentally ill among its population.^22^ Ironically, it was not far from the actual statistics of a total of 2 827 admissions into mental hospitals and 41 136 attendances at psychiatric centres.^23^ Against the expanding psychiatric service, a sense of mental health crisis emerged in media and converged with neurasthenia as a common expression of mental distress.

From the 1960s, beyond the gaze of the official and medical professionals, neurasthenia spread like wildfire in the press and became a lay term to describe a common urban illness in Hong Kong. As early as the early twentieth century China, neurasthenia already became prevalent among Chinese in urban centres. Intellectuals from different schools of psychiatry and psychology associated the prevalence of neurasthenia with national survival and modernisation in the face of imperialist encroachment.^24^ Western-trained psychiatrists and traditional healers both popularized the term neurasthenia in the early twentieth century as one of the most common way to express distress.^25^ The mass migration of Chinese refugees into Hong Kong brought similar culture and expression about neurasthenia. Following the expanding psychiatric service, neurasthenia was deracialised and converged with abnormal behaviours, murder, suicide or in general mental distress in media. Medical columns that introduced readers to new and prevalent diseases placed much emphasis on neurasthenia. With the startling title ‘Urban Epidemic’, a medical column in the local newspapers *Wai Kiu Yat Po* (華僑日報) summarised the prevalence of neurasthenia in 1960: ‘living in Hong Kong such a crowded urban city, the hustling environment and intensive work made most of the citizens more or less neurasthenic’. With influences from Freudian theory, the article indicated that the core of neurasthenia was anxiety that lived in subconsciousness, sometimes caused by childhood experiences and triggered by random changes in life. The nervousness was mental and physical when people were exhausted from work and family issues. Fortunately, neurasthenia was curable with rest cure or simple solutions, such as going for a walk, watching a movie or spending the weekend in the suburbs to freshen up the mind.^26^

Another medical column in 1970 took neurasthenia more seriously. In a threatening tone, the article described neurasthenia as an invisible illness that caused pain in patients from head to toe without any pathological changes in the body. The patients looked like ordinary people whose physical symptoms could not even be detected by a thorough physical examination in the hospital. To the author, neurasthenia was the by-product of modernisation and urbanisation. As a result, neurasthenia was uncommon in prewar Hong Kong, when people lived in a simpler way with fewer material needs and fewer troubles in life. The article defined neurasthenia as a mental health problem – once the patients identified their problem as psychological but not somatic, their symptoms would be relieved and they began to cure. It further elevated the prevalence of neurasthenia in Hong Kong to a social problem. The patients’ anxiety could result from social injustice, an unreasonable education system, an unfair working environment, false medical advertisements or a lack of recreational activities and entertainment.^27^ Although these medical columns introduced varying theories and approaches to understanding neurasthenia, they similarly stressed the ubiquity of neurasthenia.

In many respects, neurasthenia was a product of its time. During the late 1940s, the colonial government was in a dire situation, facing the aftermath of war, the prevalence of epidemic, the surge of refugees from mainland China, and the subsequent shortage of housing and food.^28^ From the 1950s, the relocation of Shanghai and Guangdong industrialists, the United States’ trade embargo against PRC China, and the influx of refugees from mainland China transformed Hong Kong from an entrepôt for China to an industrialised city. By 1966, two-fifths of the labour force worked in manufacturing.^29^ Nonetheless, most workers suffered from extreme working conditions, labouring ten to twelve hours per day and seven days a week with only four annual holidays. With no medical insurance and paid sick leave, workers had to depend on family members to pay for medicine, and less than 20 percent of factory workers had access to healthcare in the workplace.^30^ Meanwhile, rapid urbanisation took place. The influx of refugees forced a reluctant colonial government to alleviate the immense population pressures by investing in town planning. In the 1950s, the government developed the first blueprint for urban planning, established the experimental New Town in Kwun Tong, built resettlements blocks to provide emergency housing, and dispersed communities into different industrial and commercial areas. In the 1960s and 1970s, urbanisation took up much faster with the construction of satellite cities in the New Territories, the expansion of transport networks and the emergence of the first private housing estate.^31^ The transformative changes in political, economic and social life translated into mass anxiety over neurasthenia: being impotent, sick and uncompetitive in a society industrialising and urbanising at an astonishing speed.

The popularity of neurasthenia among laypeople led to a peculiar situation. Writers with Western education and training often contested neurasthenia’s association with Chinese medicine and did not regard it as a category of a formal mental illness. In 1951, Yu, a commentator, published an article titled ‘Rectify the Misconceptions about Neurasthenia’ that rejected the overuse of ‘neurasthenia’ in what he considered unreliable and harmful medical advertisements. To him, neurasthenia was a mild syndrome of mental illness that could only be treated by a psychiatrist and not by proprietary medicines seen in local advertisements.^32^ Contemporary survey in the 1980s indicates that due to neurasthenia’s popularity, psychiatrists in Hong Kong and Taiwan frequently used it in communication with patients but discarded the term as a diagnostic category in paperwork and official documents.^33^ At the same time, general practitioners with relatively limited specialised knowledge in psychiatry continued to use neurasthenia to diagnose patients, especially since most patients assumed neurasthenia to be a somatic illness and often consulted general practitioners instead of psychiatrists.^34^ The tensions between the psy-professions and traditional Chinese medicine began to emerge. It then raises a critical question: on what grounds did practitioners of Chinese medicine claim themselves to be an expert in neurasthenia, a disease category that did not exist in Chinese medical classics?

## Re-interpreting neurasthenia in everyday life

Chinese medicine has always been an evolving tradition that effectively incorporated biomedical idioms. Historian Sean Hsiang-lin Lei convincingly argues that, against the nationalist government’s attempt to abolish Chinese medicine in the 1920s, traditional Chinese doctors unified the fragmentary sections of Chinese medicine and attempted to ‘scientise’ (kexuehua) the field. As a result, a new type of ‘mongrel medicine’ emerged where Chinese medicine practitioners stressed the therapeutic values of Chinese medicine but drew on biomedical principles, such as germ theory, to justify it.^35^ In this way, traditional medicine could respond to new theories and diseases that do not correspond directly with the Chinese medical classics. In the realm of mind and brain, the most distinct difference between Chinese medicine and biomedicine was the conception of cognition. While Chinese medicine posited that the brain played a minor role in bodily function, biomedicine suggested the brain was the sources of mental processes. Nonetheless, Chinese practitioners attempted to assimilate the biomedical theory of the brain as the primary organ of cognition from the Republican period (1911–1949). For instance, Zong Zihe correlated the heart (the basis of cognition in Chinese medicine) with the brain and posited that the brain served as a vessel to store the intellect and ingenuity produced by the heart. In doing so, these practitioners utilised biomedical knowledge to enhance, justify and modernise the practice of Chinese medicine.^36^

Hong Kong constitutes an important arena to examine the history of Chinese medicine. Traditional Chinese medicine was already a favourable healing option before the 1940s when the Tung Wah Hospital, a Chinese-funded and -operated hospital, offered Chinese medicine services to local communities.^37^ The postwar period witnessed a booming field of traditional Chinese medicine in Hong Kong, and mass migration brought practitioners and entrepreneurs from Shanghai and Guangzhou to Hong Kong. A newspapers survey showed that practitioners increased thirty times from thirty-eight in 1948 to 821 in 1960, while the number of companies that manufactured and processed Chinese herbs and proprietary medicines doubled from 193 in 1948 to 487 in 1960. ^38^ According to a government survey in 1969, the numbers of Chinese practitioners were 4 506, and Western-trained doctors were 2 317.^39^ Although the Hong Kong government only provided biomedical services in the public sector, religious healing, Chinese medicine and biomedicine coexisted in the 1960s and 1970s. Patients would alternate between medications based on its efficiency and personal need.^40^ A survey conducted in 1966 showed that around half of the patients who attended government hospitals had experiences consulting traditional medicine, showing the apparent acceptance of Chinese medicine in Hong Kong.^41^ Psychiatrists’ research in 1980s Hong Kong also highlighted that, before reaching the psychiatric outpatient clinics, patients would consult different medical resources and networks, including friends, family, self-care, Western doctors, traditional Chinese doctors and religious healing.^42^ Integrating all these insights, the following section examines how Chinese practitioners and medical entrepreneurs strategically rebranded neurasthenia to cater to a booming market of caring for the mind.

To many Chinese medical practitioners, neurasthenia was not a new disease invented by Western psychiatrists but simply a different expression of existing disease categories in Chinese medicine. In the self-care booklets published by practitioners in Hong Kong, they succinctly presented neurasthenia in simple language to the readers. For example, Chen Curren (陳存仁), a reputable practitioner who relocated from Shanghai to Hong Kong in the 1950s, suggested that neurasthenia could be expressed in the form of fever, being sceptical and oversensitive (狐惑病), convergence of hundred diseases from the whole body (百合病) or hysteria (臟躁症) according to Chinese medical classics. Yet, according to Chen, most cases of neurasthenia were identified as the lack of vital energy (氣虛症) that resulted in a weakening nervous system. Neurasthenia was also a disease overlapping with other common diseases, such as anaemia, chronic diseases, hereditary or congenital diseases or overindulgence in sex. ^43^ Like many practitioners in the Republican period, Chen reinterpreted neurasthenia by borrowing traditional medical idioms about mad symptoms that originated from multiple sources, including the lack of vital energy (qi, 氣), accumulation of mucous, demonic possession, excess of emotion, overuse of the cognitive faculties and stimulation from the external environment.^44^

Diet was a standard therapy to treat neurasthenia in Hong Kong. Chen Cunren suggested food as a daily treatment; one could enhance organs by consuming similar organs from animals (以形補形) in which the brain, kidney and heart were related to the function of the mind in Chinese medical theory.^45^ For instance, Chen advised his patients to consume the brain, heart and spleen from pigs. According to Chen, the pig’s spleen was rich in fat and protein and therefore improved brain and kidney function, while pig’s heart, consumed with cinnabar, served as a mild tranquiliser for patients with mild syndromes. Pig’s brain was full of protein, fat, potassium and vitamin to increase child intelligence and growth and enhance the memory performance of the elder.^46^ The emphasis on food and diet was Chen’s deliberate and calculated decision to attract Cantonese consumers, a significant population in Hong Kong who had a keen interest in curing the body through daily food consumption. In the preface of his thousand-page book on diet, he listed the importance of food therapy – namely, it was cheap, convenient and effective. ^47^ By highlighting how diet could mitigate everyday stress, Chen targeted readers who could not afford biomedicine or were hesitant to consult medical professionals.

Qigong and taiji were also useful therapies for neurasthenia. To train and cure the organs and nervous system, Yiu suggests that qigong could train patients’ attention on specific body parts through breathing with different postures – standing, sitting or kneeling. Although scientists still failed to understand why and how qigong worked, Yiu speculated that, through breathing, the mind could enter a stage of peace and calmness, relaxing the cerebral cortex. A round of treatment would include three to five times per day and continue for two to three months.^48^ Other practitioners also took taiji, a sport that required slow motion and breathing, as a therapy for neurasthenia that could concentrate the mind, like meditation to improve overall health. Taiji and qigong both served the purpose of training the mind and thereby soothing the nervous system. ^49^ Unlike diet that could be done individually, these booklets often suggested consulting specific practitioners for techniques of qigong and taiji in which minor adjustments and details could affect the overall therapeutic result. ^50^

Their publications similarly ascribed neurasthenia to socioeconomic changes and, in particular, the rapid urbanisation and industrialisation in Hong Kong. Such an approach centred on the ambiguity of neurasthenia and helped them target a broad range of patients, including workers with multiple jobs, authors who are overworked at night, intellectuals with an imbalance of nutrition, students exhausted from schoolwork or basically, people who suffered from the hardship of work and life. They commonly advised general rules for these patients: reduce workload and working hours, sleep more than eight hours, avoid working at night and be less emotional.

To these Chinese practitioners, being scientifically sound was essential to promote their treatment. Yiu incorporated neuroscience theories to explain how the cerebral cortex connected different organs and commanded the body. To him, diseases on specific organs were not isolated but a reflection of pathological change in the entire body, corresponding with the holistic approach in Chinese medicine. In this sense, the patients should not individually isolate the neurasthenic syndromes, such as insomnia or headache, but approach the illness from its root. ^51^ These practitioners also paid attention to contemporary theories about mental health. For instance, Chen correlated the concept of mental hygiene with Chinese medical theories. Not surprisingly, as a well-known Chinese practitioner in Shanghai, Chen came across the concept of mental hygiene popular in America and the 1920s and 1930s Shanghai.^52^ He viewed mental hygiene as the primary way of preventing mental illness but highlighted how it corresponded with Chinese medical theories of diseases – the internal cause and external pathogenic causes of diseases. In particular, internal pathogenic causes, according to Chen, referred to worry, overthinking, anger and stress caused by urban life. He criticised biomedicine’s obsession with bacteria and neglect of the Chinese medical theories, which articulated the concept of mental hygiene even better than the Western scientific theories.^53^ Through publication and various strategies, these practitioners made themselves an expert of neurasthenia at the time when psychiatrists abandoned the term.

## Commercialising urban neurasthenia

While practitioners laid the theoretical groundwork to incorporate neurasthenia into practice and develop various treatments, manufacturers and retailers of proprietary medicine further commercialised neurasthenia. Recent work on pharmaceutical companies aptly highlight the importance of viewing medicine as a commodity. It demonstrates the pharmaceutical industry, driven by state and business interests, created ‘imagined space for and spread knowledge of proper medicinal consumption’.^54^ In particular, patent medicine had its specific role in the medical marketplace. Consumer’s decision to purchase proprietary medicine involved a complex interplay of factors, including consumerism, efficiency, accessibility and therapeutic value.^55^ The marketing and sale of proprietary medicine, as this section demonstrates, played a crucial role in shaping consumer’s decision and understanding of neurasthenia.

Advertisements of antineurasthenia products and treatments first emerged in the 1920s China. These products were largely popular among the elite class because advertisements often depicted the sufferers as ‘a modern man in a Western suit’ and reinforced the identity of the elite in times of warfare, revolution and imperialism.^56^ Not only physicians of Chinese medicine but also emerging psychiatric professionals advertised their treatments, including rehabilitation, tonics and proprietary medicine. Meanwhile, the boundaries of neurasthenia also expanded and converged with kidney deficiency (腎虧) due to its similar symptoms. Chinese physicians believed the kidney was in charge of producing, circulating and storing vital energy (qi, 氣), and sexual intercourse would disperse qi, resulting in an overall weakening body and health.^57^ Against the broader anxiety of impotence, advertisements in modern China also developed new products specifically for sexual neurasthenia (性神經衰弱) as a result of masturbation and addiction to sex.^58^ Nonetheless, the meanings of neurasthenia remained flexible and contingent on how retailers advertised their products. In such light, postwar Hong Kong presented a different case: Chinese proprietary medicine dominated the market and neurasthenia was no longer limited to male intellectuals.

Against the broader backdrop of urbanisation, industrialisation and abandonment of neurasthenia as a clinical label, the retailers of Chinese medicine maintained the popularity neurasthenia. They moved away from the notion of modernisation to describe neurasthenia as a ubiquitous urban illness. Advertisements of antistress products swamped the city, ranging from supporting belts, massage chairs, brain tonics, royal jelly, blood-replenishing potions, ginseng extract and antler essence. One advertisement captures the fluid meanings of neurasthenia with an eye-catching title, ‘Are you neurasthenic?’ and then introduced the disease:

Neurasthenia was a comprehensive noun, especially in the urban area where the patients are everywhere. According to a survey in foreign countries, the more prosperous a city is, the more neurasthenic patients. In short, living in a complex and bustling society, individuals cannot resist external stimulants in which the nervous system would receive and sense happiness, anger, or sadness. In addition, gatherings, festivals, and entertainment at night deprives the essential part of human life to rest – sleeping… the patient would normalise the constant stress and suffer from insomnia due to hypertension in the nervous system. Many people underestimate the severity of insomnia – we would not forget the cases of suicides in newspapers: the most severe neurasthenic patients jumped off from the building or killed themselves with poison. Sedative and tranquillisers will only control the patients’ emotions temporarily. It cannot eliminate the problem, and consumption of these drugs, in the long term, is as harmful as drug abuse.^59^

According to this advertisement, the only solution was the medicine *Ginroymin,* which could replenish the nervous system, help handle schoolwork, improve work efficiency and cleanse the mind. Although it claimed to develop from a German pharmaceutical company’s recipe, *Ginroymin* was possibly traditional Chinese medicine repackaged to look like modern medicine, being a mixture of royal jelly, ginseng and other nutrients.^60^ This lengthy quotation is representative in the sense that other entrepreneurs of proprietary medicine used similar advertising strategies to promote their products: invoking both Western and Chinese medical theories, devaluing sedatives and tranquilisers introduced by psychiatrists, highlighting the prevalence of urban stress and associating insomnia with suicide. Advertisements shared a standard view about the causes of neurasthenia: pressure from work and the rapid pace of urban life triggered neurasthenia, and therefore, all people in Hong Kong were exposed to the danger of being neurasthenic. At times, it could be a synonym of mental illness, a way to describe exhaustion from overwork, a bodily reaction toward pressure or even mental collapse in response to rapid societal change. Other advertisements were often in shorter form, directly listing out syndromes related to neurasthenia. These advertisements served the same purpose to instruct the patients to self-diagnose and convince them to purchase the products. Eventually, neurasthenia became a catch-all of urban illness in postwar Hong Kong.

Meanwhile, Western pharmaceutical products did not achieve commercial success. The 1950s and 1960s have often be characterised as the golden era for tranquilisers and antidepressants in Europe–American contexts, in which pharmaceutical companies successfully commercialised mild mental distress and turned consuming medicine into a daily habit for the masses. But, the success of tranquilisers and antidepressants did not spread to Hong Kong. In the late 1950s, the sale of tranquilisers, such as Miltown, quickly fell under the scrutiny of the colonial government because of its potential ‘habit-forming’ nature. Tranquilisers were dangerous, especially in the 1950s when the colonial government launched an antinarcotic war to ban opium and heroin entirely. In 1957, the Pharmacy Board first prohibited the sales of ‘Relaxa-Tabs’ that contained tranquilisers (carbromalum and bromvalerylurea) as a remedy for sleeplessness due to worry, anxiety, grief, overwork, excitement and so on, which resulted in six attempted suicides, two of which were successful.^61^ Alarmed by the side effects, the government added new clauses to the Pharmacy and Poisons Ordinance that regulated the sale of tranquilisers only by prescription.^62^ At the same time, the *Undesirable Medical Advertisements Ordinance* strictly limited pharmaceutical companies’ ability to promote medicine claiming to cure symptoms such as insomnia, fatigue and other common illnesses.^63^ As a result, tranquilisers circulated in the market on a limited scale in the 1960s and 1970s.

Chinese proprietary medicine had a distinct edge over tranquilisers because of its exemption from the two ordinances. During the legislation process, the colonial government deliberately avoided confrontation with Chinese medicine, a custom widely used by the Chinese communities. In the words of anthropologist Marjorie Topely, legislating against Chinese customs was seen as an unwise, unrealistic and a dangerous move. The colonial government, therefore, neither acknowledged nor regulated Chinese medicine. Instead, it attempted to ‘entice’ the population by promoting and subsidising Western medicine to demonstrate the advances of Western medicine.^64^ Although physicians often complained about the unfair treatment, the government turned a blind eye to the prevalent advertisements of Chinese medicine in most cases. The colonial government’s ambivalent attitude opened up a big market to companies of Chinese proprietary medicine.

Due to the government’s lax restrictions, Chinese pharmaceutical companies could brand their products towards neurasthenia more flexibly than most Western pharmaceutical products. One example is the *Young Yum Pills* (養陰丸), a best-seller medicine from Wai Yuen Tong established in Hong Kong in 1930. In 1954, an advertisement described *Young Yum Pills* as a quick fix to only two syndromes – coughing and phlegm.^65^ Another advertisement of the same product in 1964 contained an expanding list of over ten syndromes: coughing, phlegm, digestion, replenishing blood, reinforcing *qi*, warming yin (陰), fatigue and neurasthenia.^66^ In light of neurasthenia’s overlapping boundaries with other illnesses, the companies seldom promoted their products as a cure specific to neurasthenia per se but rather to a range of symptoms, including relieving nervous stress, improving blood flow, enhancing memory or rejuvenating youth. A manual of Chinese proprietary medicine in Hong Kong quoted at least 24 medicines available in the market that implicated neurasthenia, many of which focused on calming the mind (安神). The category of sedative and tranquilising formulas (安神劑)^67^ corroborated with the Chinese medical theory of nourishing blood and replenishing the heart to soothe mental disturbance caused by deficiencies of yin. Another category of tonic formulas (補益劑) treated similar symptoms of general ill health, including insomnia, night sweats and amnesia due to lack of qi and deficiency in the kidney. Around ninety types of medicine listed under tonic formulas were quoted in the manual for treating neurasthenia-related symptoms.^68^ In this sense, the ambiguity of neurasthenia gave much space to practitioners to theorise the illness and to the entrepreneurs of proprietary medicine to market their products strategically.

The greatest advantage of Chinese proprietary medicine was its cost and convenience. Here a comparison with Western pharmacies would help clarify how patients made their medical decisions. First, unlike tranquilisers which could only be obtained by prescription, Chinese proprietary medicine was readily made and accessible, often in a box of pills and sold at numerous Chinese-style pharmacies. Second, Chinese proprietary medicine was much cheaper. By the 1960s, the price of antineurasthenic medicine ranged from $2.50 to $10, depending on the reputation of the companies and the materials used in the medication. Some included 300 and even 500 tablets per package. Given that the average daily wages of industrial workers were $5.53 in 1958 and $11.20 in 1967,^69^ the Chinese proprietary medicine was affordable to most households. It posed a sharp contrast with tranquilisers, sedatives and antidepressants, such as eight doses (enough for a week) of Miltown was sold at $6.35 by 1973.^70^ The fact that proprietary medicine did not need a prescription and was easily accessible undoubtedly shaped how consumers made their decisions. Table A1 summarises the prices and types of proprietary medicine extracted from available sales manuals.

With restrictions on antidepressant advertisements, Chinese proprietary medicine gained a particular edge. Retailors of proprietary medicine were exempted from the colonial legislation. They not only popularized and diffused knowledge about the illness but also instructed the readers to self-diagnosis and convinced them to purchase products for self-medication.^71^ Although the mass anxiety of fatigue and nervousness was inseparable from their expanding commercial interests, the extent to which consumers subscribed to such advertising strategies remains unexplored. The following section examines patients’ responses to neurasthenia by analysing patient-written narratives reprinted in medical columns.

## Experiencing neurasthenia

Medical columns by practitioners of Chinese medicine became popular in postwar Hong Kong. These medical columns were crucial channels for ordinary people to access information related to health and medicine, especially when medical resources were limited in the postwar period.^72^ The newspapers also invited practitioners of Chinese medicine to write on various subjects, such as diet, seasonal epidemics or health in general. These practitioners, such as Chen and Yiu mentioned in the earlier section, built a mutually beneficial relationship with the press, promoted their treatment and medication and enjoyed great commercial success.^73^ Among all of the columns, this section looks at the column *Medical Consultant* (醫學顧問) in *Wen Wei Po* (文匯報), a local newspaper sponsored by the Chinese Communists in Hong Kong, offers a glimpse into the public perception of neurasthenia.^74^ The column followed the earlier model of *Shanghai Wen Wei Po* and invited medical experts to answer readers’ letters in which the ‘patients’, who submitted letters to the newspapers, described their background and details of their illness and asked for advice from the ‘expert’. The column would then publish the responses from experts, giving basic diagnoses based on the description and offering guidance to the patients. The column also occasionally printed out letters from readers, showing the patients’ self-narratives about their illness and mental distress. Available archives show that 417 out of the 5 878 published responses were about neurasthenia between 1948 and 1987, with most (350) largely congregated in the 1960s and 1970s. These materials not only address readers who submitted letters but also readers who shared similar concerns. Most importantly, the *Wen Wei Po* in Hong Kong was one of the widely read newspapers with a daily circulation of 33 500 in May 1967, compared to 81 300 copies by the popular *New Evening Post.* ^75^ This study suggests these patients letters show how laypeople understood, encountered and coped with their neurasthenic symptoms.

To most patients, neurasthenia was often a confusing illness. For example, a patient wrote, in 1963 and described his symptoms in this way: he had been sick for six years with severe dizziness, suffered from headaches, blurry vision and unusual heart beating. He underwent multiple treatments and received different diagnoses. A Western medicine doctor checked the blood and urine sample, did an X-ray of his lungs and said it was insomnia and neurasthenia; while a practitioner of Chinese medicine, by contrast, understood it as a general state of feebleness.^76^ Another patient, a 15-year-old student, went to doctors for difficulties in breathing. He was diagnosed as tracheitis, issues of blood circulation and neurasthenia by different Western doctors. He also tried Chinese medicine. A practitioner of Chinese medicine suggested the lack of qi, while a doctor who combined Chinese and Western medicine commented his case as neurasthenia.^77^ By consulting different doctors, these patients received different and sometimes conflicting diagnoses – neurasthenia, heart disease, asthma, anaemia, shenkui, weak immune system, rhinitis or other illnesses that shared a similar set of syndromes: headache, dizziness, heart beating, fatigue and insomnia. The protean nature of neurasthenia to a certain extent explained its prevalence that one could easily self-identify or be diagnosed as neurasthenic.

Among all the letters examined for this article, self-diagnosis and self-medication were extremely common. For example, a male patient around 30 years old felt dizzy and had a headache after overnight work and masturbation. Without consulting any doctor, he consumed supplements and proprietary medicine every day, including three pills of American ginseng hormone supplement, thirty drops of ginseng extract and forty drops of antler essence (鹿茸精). The expert concluded his case as neurasthenia and advised him to consume one-third of the current dose.^78^ Another patient, after being diagnosed with neurasthenia by a Chinese practitioner and Western-trained doctor, did not continue the treatment; instead, he bought Chinese and Western proprietary medicine advertised in newspapers.^79^ These letters seeking advice did not suggest a straightforward doctor–patient relationship in which the patients always complied with the instructions and advice of the corresponding doctor. For instance, many who had consulted the doctors would also write to the column to verify their symptoms or question an earlier diagnosis. Some patients consulted several Chinese doctors and Western medicine and tried several treatments, including electrotherapy, acupuncture, taiji, qigong, proprietary medicine and herbal medicine that worked on them.

The letters, fundamentally, reflected patients’ self-understanding of their bodily suffering, offering important insights into public perceptions and responses towards neurasthenia. One letter, in 1949, was a detailed self-analysis about a patient’s mental distress. The author, who escaped to Hong Kong in the 1940s to avoid warfare between the communist and nationalist governments, suffered from exhaustion and weakening eyesight. He identified himself as ‘neurasthenic’ and speculated about the origins of his illness – the partially grown nervous systems, his child-like size of sexual organs and military training in the past that eventually triggered his symptoms. He tried various treatments, including vitamin B and sedatives, with ineffective results. To him, neurasthenia was the ‘illness of the century’, and he attributed his depressed mood to the broader social, political and economic changes in modern China. The expert agreed with most of his observation, clarified some misconceptions and gave some general advice.^80^ In these correspondences, the expert largely depended on patients’ testimony and responded to their problems based on patients’ interpretation of symptoms.

Most importantly, neurasthenia was not considered a formal mental illness in the eyes of patients. The expanding psychiatric services from the 1960s admitted unprecedented numbers of patients into hospitals and media often portrayed mentally ill patients as dangerous and unpredictable. One example of the stigma attached to mental illness was public opposition towards the construction of halfway houses for ex-mental patients in residential area in the 1970s.^81^ Unlike the ‘real’ mental illness that psychiatrists treated in a hospital setting, neurasthenia was disassociated from mental illness and thus less stigmatised. Although the neurasthenic identity was no longer seen as a privilege for the educated and elite class, the fact that the patients publicly discussed and shared personal symptoms of neurasthenia was unusual. Also, patients often considered neurasthenia a somatic problem instead of a psychiatric one, which explains their preference in medical decisions for treating neurasthenia. As many anthropologists have shown, neurasthenia as a medical label avoided the shame and guilt associated with insanity. Patients often considered a psychiatrist the least favourable option compared to general practitioners, traditional Chinese medical doctors and religious healers.^82^ That explains why experts in the column often responded to and reaffirmed the patients that they were only neurasthenic, differentiating minor mental disturbance from acute psychosis or schizophrenia that warranted hospitalisation. To the patients, neurasthenia sheds a different light on interpreting their illness and decide their medication.

The response from the medical experts also illustrates both the Chinese and Western doctors’ understandings of neurasthenia. Wen Wei Po had considerable connection with the PRC and served as a medium for the Communist state to win the hearts and minds of the public in Hong Kong.^83^ For the column *Medical Consultant,* Wen Wei Po hired a team of medical practitioners in Hong Kong and China. Available evidence indicates that one of the ‘medical experts’ was Dung Ngok Lam (董岳琳), a leading practitioner of Chinese medicine in Guangdong.^84^ From 1951 to 1971, the medical experts often directed the readers to consume ginseng and Chinese proprietary medicine to cure their syndromes. They also listed a prescription for Chinese herbs for self-medication^85^ and referred the patients to specific acupuncturists in Hong Kong.^86^ After 1971, the experts showed a stronger tendency to support biomedicine. They did not refrain from using the term neurasthenia and offered a prescription of sedatives, like Valium and Librium. ^87^ The expert’s response to mental health issues often suggested the patients consult psychiatrists in possible cases of schizophrenia.^88^ Nonetheless, it would be misleading to characterise Chinese and biomedicine in binary terms regarding their treatments and diagnosis. For example, for those supported Chinese medicine, they smoothly incorporated the biomedical language into diagnosis. They would suggest a full-body check-up in the hospital to look for possible causes beyond the paradigm of Chinese medicine, such as parasites.^89^ The experts – whether with different backgrounds in Chinese or biomedicine – offered similar generic suggestions to neurasthenic patients: namely, that readers should pursue a disciplined lifestyle, eat and sleep regularly, maintain a positive mindset and engage in sports. These correspondences show that, beyond the gaze of professional psychiatry, patients could possibly come across similar advices from general practitioners and traditional Chinese doctors.

The patients’ letters presented a complex scene of the medical marketplace for curing neurasthenia. Historians in the past decades showed how the patient’s view is essential to understand the history of medicine from below. Patients could freely choose and try different medications based on their needs, such as magical healing, patent medicine or herbal medicine, in which professional practitioners played a marginal role in making medical decisions. ^90^ Historian Wen-Ji Wang examines the diaries of Gu Jiegang (顧頡剛), an imminent intellectual in Republican China, who tried a number of treatments, compared their efficiency on his body and documented his decades-long therapeutic journey.^91^ In a similar vein, a critical analysis of these letters reveals that the patients were exposed to a diverse array of treatments and had to make the medical decisions. From the 1960s, the expanding psychiatric service led to an unprecedented number of patients admitted into or attended psychiatric facilities, while traditional Chinese medicine filled the vacuum in medical marketplace of neurasthenia and exploited the legal loopholes to promote their treatments in media. The patients, as shown in the letters, were more exposed to traditional Chinese medicine, which were more accessible, culturally familiar and cheaper. But they did not blindly support to either Western psychiatry or traditional Chinese medicine. They experimented with different treatments, chose multiple medications, observed their bodily changes, evaluated the treatment’s therapeutic value and even challenged the doctor’s diagnosis.

Nonetheless, it is impossible to determine whether the readers indeed agreed with the medical advice, just as they disagreed with the doctors and practitioners they consulted. Also, since the column was open to public, some letters could be advertisements from the pharmaceutical companies or practitioners to promote their cure. Fundamentally, the patients’ letters selected here were part of their therapeutic journey to search for a cure for their symptoms, offering fragmentary yet essential ways to understand how patients encountered, interpreted and responded to bodily suffering. From their experiences, general practitioners, physicians of Chinese medicine, proprietary medicine and sedatives or tranquiliser did not guarantee recovery in treating neurasthenia.

## Conclusion

This study situates the persistence of neurasthenia in the social history of mental health during the mid and late twentieth-century Hong Kong. Following psychiatrists discarding of the term neurasthenia due to its ineffectiveness and impreciseness in the 1950s, Chinese medicine practitioners and Chinese pharmaceutical companies based in Hong Kong embraced its ‘useful ambiguity’. They skilfully positioned their treatments and products to fill in the gap in the medical marketplace – presenting in a familiar language, adopting a less invasive approach through daily care and blending Chinese medicine with biomedical theories. Given that Chinese medicine has constantly evolved, it is not surprising to see that practitioners could reinvent themselves to address disease categories that did not exist in the Chinese medical classics, such as neurasthenia and, later, depression. Thus, the story of neurasthenia was not simply about a disease label. Fundamentally, it is about how patients chose to interpret their illness and selected medications in a diverse array of treatments and products.

Focusing on the medical marketplace and patients’ narratives, this study scrutinises how neurasthenia became a specialty in traditional Chinese medicine and persisted as the most popular term to describe minor mental distress. Psychiatric and anthropological research of neurasthenia from the 1970s enquired about the nature of mental illness in Asia: whether neurasthenia was another form of depression or whether traditional Chinese medicine was effective in treating mental distress. By contrast, this study suggests it is a more complex historical dynamics. When psychiatrists abandoned neurasthenia as a clinical label, practitioners and retailers of Chinese medicine maintained its popularity and made it a speciality in Chinese medicine. As such, an historical analysis of neurasthenia invites further research that might historicise other culturally specific disorders in Western psychiatry, often termed as ‘cultural bound syndromes’,^92^ in order to explore the tensions between traditional medicine and Western psychiatry.

Finally, this case study of Hong Kong bears relevance to examining the widespread use of neurasthenia as a diagnostic entity in twentieth-century Asia. Anthropological and psychiatric research proved the popularity of neurasthenia among the public in the Chinese-speaking world. In particular, Hong Kong was tightly connected with Chinese migrants in Southeast Asia and Taiwan. Similar publications, advertisements and proprietary medicine circulated between these places. For instance, newspapers from Singapore in 1973 summarised a list of reading materials about neurasthenia, and eight of ten books originated from Hong Kong.^93^ Publications from practitioners of Chinese medicine, such as Chen Cunren, also republished widely among Chinese newspapers in Singapore, Thailand, Vietnam, Indonesia the Philippines, and the United States in multiple languages.^94^ Hong Kong also shared similar aspects with other postcolonial states regarding the relatively lax regulation of traditional Chinese medicine in Malaysia and Singapore.^95^ The complexity and prevalence of neurasthenia in Asia invites further historical study.

## Footnotes

1

George M. Beard , ‘Neurasthenia, or Nervous Exhaustion’, Boston Medical and Surgical Journal, 3, 13 (1869), 217–21.

2

David G. Schuster , Neurasthenic Nation: America’s Search for Health, Happiness, and Comfort, 1869–1920 (New Brunswick: Rutgers University Press, 2011), 1 .

3

Arthur Kleinman , Social Origins of Distress and Disease: Depression, Neurasthenia, and Pain in Modern China (New Haven; London: Yale University Press, 1986), 15–20 .

4

Tsung-Yi Lin , ‘Neurasthenia Revisited: Its Place in Modern Psychiatry’, Culture, Medicine and Psychiatry, 13 (1989), 111; 12310.1007/BF022206562766788.

5

Char-nie Chen , ‘Public Education & Promotion of Mental Health’, in Tai-pin Khoo (ed.), Aspects of Mental Health Care: Hong Kong 1981 (Hong Kong: Mental Health Association of Hong Kong, 1981), 38 .

6 The most well-known work of somatization and neurasthenia is Kleinman, *op. cit.* (note 3). The study of somatization includes a huge body of literature and here only includes the most relevant one; see Norma C. Ware and Mitchell G. Weiss , ‘Neurasthenia and the Social Construction of Psychiatric Knowledge’, Transcultural Psychiatric Research Review, 31, 2 (1994), 101–24; Thomas J. Csordas (ed), Embodiment and Experience: The Existential Ground of Culture and Self (Cambridge: Cambridge University Press, 1994); Laurence J. Kirmayer , ‘Culture, Affect and Somatization’, Transcultural Psychiatric Research Review, 21, 3 (1984), 159–88; Sing Lee , ‘Diagnosis Postponed: Shenjing Shuairuo and the Transformation of Psychiatry in Post-Mao China’, Culture, Medicine and Psychiatry 23 (1999), 349–8010572739 10.1023/a:1005586301895; Allen Tran , A Life of Worry: Politics, Mental Health, and Vietnam’s Age of Anxiety (Berkeley, CA: University of California Press, 2023).

7

Warwick Anderson , Colonial Pathologies: American Tropical Medicine, Race, and Hygiene in the Philippines (Durham: Duke University Press, 2006), 132–3. For the history of neurasthenia in the colonial context, please see: Anna Crozier ‘What Was Tropical about Tropical Neurasthenia? The Utility of the Diagnosis in the Management of British East Africa’, Journal of the History of Medicine and Allied Sciences, 64, 4 (2009), 518–4819531548 10.1093/jhmas/jrp017; Will Jackson , Madness and Marginality: The Lives of Kenya’s White Insane (Manchester: Manchester University Press, 2013); Laurence Monnais , ‘Colonised and Neurasthenic: From the Appropriation of a Word to the Reality of a Malaise De Civilisation in Urban French Vietnam’, Health and History, 14, 1 (2012), 121–4223066605 10.5401/healthhist.14.1.0121; Yolana Pringle , ‘Neurasthenia at Mengo Hospital, Uganda: A Case Study in Psychiatry and a Diagnosis, 1906–50’, The Journal of Imperial and Commonwealth History, 44, 2 (2016), 241–6227335533 10.1080/03086534.2015.1123975PMC4894077; Theodore Jun Yoo , It’s Madness: The Politics of Mental Health in Colonial Korea (Berkeley, CA: University of California Press, 2016).

8 Crozier, *op. cit.* (note 6), 547–8; Monnais, *op. cit.* (note 6), 140–2.

9

Wen-ji Wang , ‘Neurasthenia, Psy Sciences and the “Great Leap Forward” in Maoist China’, History of Psychiatry, 30, 4 (2019), 443–5631238740 10.1177/0957154X19859204.

10

Howard Chiang , ‘Translating Culture and Psychiatry across the Pacific: How Koro Became Culture-Bound’, History of Science, 53, 1 (2015), 102–19.

11

Wen-Ji Wang , ‘Tropical Neurasthenia or Oriental Nerves? White Breakdowns in China’, in Howard Chiang (ed.), Psychiatry and Chinese History (London: Pickering & Chatto, 2014), 126 .

12 CO129/442, Letters from G. E. Aubrey, 5 June 1917, Hong Kong Public Record Office (H.K.P.R.O hereby).

13 CO129/549/2, Form of Employment Agreements for Police Officers, 3 August 1934, H.K.P.R.O.

14 CO129/590/11, W. B. L. Monson Telegram, 25 April 1941, H.K.P.R.O.

15

Wen-Ji Wang , ‘From Nervelessness to Nervousness: Images of Chinese Mental and Nervous Illnesses in Western Medical Discourse, the 1840s–1930s’, Yonsei Journal of Medical History, 23 (2020), 48 .

16 South China Morning Post (SCMP hereby), ‘The Balkan Cloud Breaking’, 30 November 1912.

17 The data are gathered from *Hong Kong Administrative Reports* (Hong Kong: Government Printer) from 1910 to 1939.

18 Schuster, *op. cit.* (note 3), 157–8.

19

Helen F. K. Chiu , ‘Professor Pow-Meng Yap: A Giant in Psychiatry from Hong Kong’, Asia-Pacific Psychiatry, 4 (2012), 84–6.

20
*Annual Departmental Reports by the Director of Medical and Health Services* (Hong Kong: Government Printer, 1950).

21
*Annual Departmental Reports by the Director of Medical and Health Services* (Hong Kong: Government Printer, 1962), 71–2.

22 Wai Kiu Yat Po, ‘The Growing Number of Mental Health Patients: Castle Peak Hospital Became Overcrowded’ (精神病患者日有增加,青山醫院有人滿之患), 31 August 1960.

23
*Annual Departmental Reports by the Director of Medical and Health Services* (Hong Kong: Government Printer, 1966), 119.

24

Wenji Wang , ‘Neurasthenia and the Rise of Psy Disciplines in Republican China’, East Asian Science, Technology, and Society: An International Journal, 10, 2 (2016), 141–60. He also examines how specific Cold War geopolitics provided space for Taiwanese psychiatrists to present themselves as leading experts on Chinese culture and neurasthenia in the international academia; see Wen-Ji Wang , ‘Managing Chineseness: Neurasthenia and Psychiatry in Taiwan in the Second Half of the Twentieth Century’, History of Psychiatry, 33, 3 (2022), 1–16 10.1177/0957154X22108741035466754.

25 Emily Baum, ‘Choosing Cures for Mental Ills: Psychiatry and Chinese Medicine in Early 20th Century China’, *Asian Review of World Histories*, 6 (2018), 11.

26 Wah Kiu Yat Po, ‘Urban Epidemic: Neurasthenia’ (都市流行病: 神經衰弱), 31 August 1960.

27 Gam Wai (甘衛), ‘Urban Illness: Neurasthenia’ (現代都市病-神經衰弱症), *The Seventies* (七十年代), 9 (1970), 52–3.

28

John M. Carroll , Concise History of Hong Kong (Hong Kong: Hong Kong University Press, 2007), Chapter 5.

29

Steve Tsang , Modern History of Hong Kong (London: I. B. Tauris, 2004), 162 .

30

David Clayton , ‘From “Free” to “Fair” Trade: The Evolution of Labour Laws in Colonial Hong Kong, 1958–62’, Journal of Imperial and Commonwealth History, 35, 2 (2007), 267–8; 275.

31

Pui-yin Ho , Making Hong Kong: A History of Its Urban Development (Cheltenham, UK and Northampton, US: Edward Elgar Publishing Limited, 2018), 155–231 .

32 Yu-Ai Lu (余愛淥), ‘Rectify the Misconceptions about Neurasthenia’ (糾正關於神經衰弱的幾種誤解), *Wen Hui Po*, 22 December1951.

33

Fanny M. Cheung , ‘The Indigenisation of Neurasthenia in Hong Kong’, Culture, Medicine and Psychiatry, 13 (1989), 236 10.1007/BF022206642766796; Hsien Rin and Mei-gum Huang , ‘Neurasthenia as Nosological Dilemma’, Culture, Medicine and Psychiatry, 13 (1989), 220 10.1007/BF022206632766795.

34

Fanny M. Cheung , Bernard W. K. Lau and Sai-wo Wong . ‘Paths to Psychiatric Care in Hong Kong’, Culture, Medicine and Psychiatry, 8 (1984), 207–286488844 10.1007/BF00055168; Fanny M. Cheung and Bernard W. K. Lau . ‘Situational Variations in Help-Seeking Behaviour among Chinese Patient’, Comprehensive Psychiatry, 23, 3 (1982), 252–627083852 10.1016/0010-440x(82)90073-6.

35

Sean Hsaing-lin Lei , Neither Donkey nor Horse: Medicine in the Struggle over China’s Modernity (Chicago: The University of Chicago Press, 2014).

36

Emily Baum , The Invention of Madness: State, Society, and the Insane in Modern China (Chicago, London: The University of Chicago Press, 2018), 160–4.

37 Yang Xiangyin (楊祥銀), ‘Colonial Power and Medical Space: Transformation of Chinese and Western Medical Services in the Tung Wah Group of Hospitals: 1894–1941’ (殖民權力與醫療空間:香港東華三院中西醫服務變遷 1894–1941) (Beijing: She Hui Ke xue wen xian chu ban she, 2018), Chapter 3.

38
*Ibid.*

39

Rance P. L. Lee , ‘Interaction between Chinese and Western Medicine in Hong Kong: Modernisation and Professional Inequality’, in Arthur Kleinman (ed.), Medicine in Chinese Cultures: Comparative Studies of Health Care in Chinese and Other Societies (Washington, DC: U.S. Department of Health, Education and Welfare, 1975), 224 .

40

Rance P. L. Lee , ‘Perceptions and Uses of Chinese Medicine among the Chinese in Hong Kong’, Culture, Medicine and Psychiatry, 4 (1980), 350 10.1007/BF000518117449412.

41

Gerald Choa , ‘Chinese Traditional Medicine and Contemporary Hong Kong’ in Marjorie Topley , Some Traditional Chinese Ideas and Conceptions in Hong Kong Social Life Today (Hong Kong: The Hong Kong Branch of the Royal Asiatic Society, 1967), 54–60 .

42 Cheung, Lau and Wong, *op. cit.* (note 29), 226–7.

43 Cunren Chen (陳存仁)*, Jinjin Youwei Tan: He Ding Ben津津有味潭: 合訂本* (Hong Kong: Hong Kong Shanghai Yin Shu Guan, 1960), 50–4.

44 For further discussion of ‘mad categories’ in traditional Chinese medicine, see Fabien Simonis , ‘Ghosts or Mucus? Medicine for Madness: New Doctrines, Therapies, and Rivalries’, in Pierre Marsone and John Lagerwey (eds.) Modern Chinese Religion I (Leiden: Brill, 2015), 601–39; Vivien Ng , Madness in Late Imperial China: From Illness to Deviance (Norman: University of Oklahoma Press, 1990).

45 Cunren Chen*, Jinjin Youwei Tan: He Ding Ben津津有味潭: 合訂本* (Hong Kong: Hong Kong Shanghai Yin Shu Guan, 1960), 54. The kidney is the organ that stores the positive *qi* in Chinese medicine. *Shenkui* is considered to be another culture-bound syndrome.

46
*Ibid.*, 8; 55–8; 250; 302; 426; 485; 487; 684.

47
*Ibid.*, 1140. At the end of his publications, he posted an advertisement inviting overseas patients to send him letters about their illnesses. He would answer the inquiry with a tailor-made medicine formula, then the patient took his reply letter and bought relevant herbs from nearby Chinese herbal shops.

48 Yiu Heung Hung (姚香雄), *How to Conquer Neurasthenia* (如何戰勝神經衰弱) (Hong Kong: Dai Guong Ced Ban Se, 1961), 99–104.

49 Leui Baak Fan (雷伯勳), *Neurasthenia and its Prevention* (神經衰弱及其防治) (Hong Kong: Dei Lei Xu Gug, 1963), 63.

50 Luk Yik Gung (陸易公), *Neurasthenia* (神經衰弱症) (Hong Kong: Lu Yigong, 1962), 29–30.

51 Yiu, *op. cit.* (note 45), 54–5.

52 Jinping Ma, ‘Remoulding the Chinese Mind–Mental Hygiene Promotion in Republican Shanghai’, (Unpublished PhD thesis: University of Warwick, 2019).

53 Chen Cunren, *Methods of Practicing Mental Hygiene* 心理衛生修養法 (Hong Kong: Shanghai Book Store, 1960).

54

Timothy M. Yang , A Medicated Empire: The Pharmaceutical Industry and Modern Japan (Ithaca; New York: Cornell University Press, 2021), 8–13 .

55 Emily Baum, ‘Health by the Bottle: The Dr. Williams’ Medicine Company and the Commodification of Wellbeing in the Liangyou Pictorial,’ in Paul Pickowicz, Shen Kuiyi and Zhang Yingjin (eds.), *Liangyou: Kaleidoscopic Modernity and the Shanghai Global Metropolis, 1926–1945.* (Leiden: Brill, 2013), 89–90.

56 Baum, *op. cit.* (note 10), 107.

57 Hugh L. Shapiro, ‘The View from A Chinese Asylum- Defining Madness in 1930s Beijing’ (Unpublished PhD thesis: Harvard University, 1995), 125–8.

58 Guoli Pi (皮國立), *History of Fatigue: Interpretation of Desire and Medical Culture in Modern Chinese and Western Medicine (1912–1949)* (虛弱史 : 近代華人中西醫學的情慾詮釋與藥品文化 1912–1949) (Taipei: Taiwan Shang Wu Yin Shu Guan Gu Fen You Xian Gong Si, 2019), 154–5.

59 ‘Are You Neurasthenic?’, *The Young Companion Pictorial,* 127 (November 1964), 37.

60
*Ibid.*

61 HKRS49-1-15, 23^rd^ Meeting of the Pharmacy Board, 22 April 1959, H.K.P.R.O.

62 HKRS49-1-15, 19^th^ Meeting of the Pharmacy Board, 15 April 1958, H.K.P.R.O.

63 HKRS49-1-15, Proposed Control the Advertising of Pharmaceutical Preparations, 8 April 1958, H.K.P.R.O.

64

Marjorie Topley , ‘Chinese and Western Medicine in Hongkong: Some Social and Cultural Determinants of Variation, Interaction and Change’, in Arthur Kleinman (ed.), Medicine in Chinese Cultures: Comparative Studies of Health Care in Chinese and Other Societies (Washington, DC: U.S. Department of Health, Education and Welfare, 1975), 255–62.

65 ‘Young Yum Pills,’ *The Young Companion Pictorial,* 1 (August 1954).

66 ‘Young Yum Pills,’ *The Young Companion Pictorial,* 120 (April 1964).

67 The translation of 安神劑 here is different from the sense of tranquilisers produced by Western pharmaceutical companies and regulated by the colonial government.

68 Heui Lok Jung (許樂中), *Manuel of Chinese Medical Prescription and Proprietary Medicine* (Hong Kong: Dak Lei Syu Guk, 1970).

69 Hong Kong Census and Statistics Department, *Hong Kong Statistics, 1947–1967* (Hong Kong, Government Printer, 1969), 65.

70
*Hong Kong Index of Medical Specialities: An Authoritative Guide to Preparations Available to Doctors, Pharmacists, and Dentists in Hong Kong Volume 1* (Singapore: Scientific Publications, 1973), 88

71 Other studies also show the roles of medical advertisements in the history of medicine. See in the context of modern China, Sherman Cochran , Chinese Medicine Men: Consumer Culture in China and Southeast Asia (Cambridge, Mass; London: Harvard University Press, 2006) and Joan Judge , Republican Lens: Gender, Visuality and Experience in the Early Chinese Periodical Press (Oakland, CA: University of California Press, 2015); for advertisements of antineurasthenic products in the United States, see Schuster, *Neurasthenic Nation.*

72

Yip Ka-che , Wong Man-kong and Leung Yuen-sang , A Documentary History of Public Health in Hong Kong (Hong Kong: The Chinese University Press, 2018), 341 .

73 Cunren Chen, *op. cit.* (note 47), 1140. At the end of his publications, he posted an advertisement that invited overseas patients to send him letters for enquiry about their illness. He would answer the enquiry with a tailor-made formula of medicine, then the patient took his reply letter and bought relevant herbs in nearby Chinese herbal shops.

74
*Medical Consultant* was part of the section of the ‘*Social University*’ (社會大學), which invited ‘experts’ in medicine, science, art, movie and other areas to answer readers’ questions about love affairs, family, career, learning, law or medicine. It became a popular column that remained active until 1987.

75

Chi-kwan Mark , ‘Everyday Propaganda: The Leftist Press and Sino-British Relations in Hong Kong, 1952–1967’, in Marco Wyss Janick Marina Schaufelbuehl and Valeria Zanier (eds.), Europe and China in the Cold War: Exchanges beyond the Bloc Logic and Sino-Soviet Split (Leiden, Boston: Brill, 2018), 163 .

76 Wen Hui Po, *Medical Consultant*, 25 September 1963.

77 Wen Hui Po, *Medical Consultant,* 8 February 1975.

78 Wen Hui Po, *Medical Consultant*, 21 November 1965.

79 Wen Hui Po, *Medical Consultant*, 31 October 1964.

80 Wen Hui Po, *Medical Consultant*, 18 May 1949.

81 Wai-Kwong Mak, ‘History, Modern Development, and Issues.’ *Journal of Sociology and Social Welfare*, 18, 2 (1991), 136.

82 Cheung, *op. cit.* (note 28), 237.

83 Mark, *op. cit.* (note 78), 166.

84 Shi Yuguang史宇廣), *The Dictionary of Practitioner of Chinese Medicine in China* (中國中醫人名字辭典) (Beijing: Zhong yi gu zhi chu ban she, 1991), 201.

85 Wen Hui Po, *Medical Consultant,* 27 April 1963.

86 Wen Hui Po, *Medical Consultant,* 4 November 1965.

87 Wen Hui Po, *Medical Consultant,* 7 July 1971; 1 July 1973; 13 October 1977; 24 July 1979.

88 Wen Hui Po, *Medical Consultant,* 16 May 1979.

89 Wen Hui Po, *Medical Consultant,* 21 December 1964.

90 Roy Porter, ‘The Patient’s View: Doing Medical History from Below,’ *Theory and Society* 14 (1985), 193–5.

91 Wen-Ji Wang, ‘Gu Jiegang and the Management of Neurasthenia in Republican China’ (知行未必合一:顧頡剛與神經衰弱的自我管理), in Pingyi Chu (祝平一) (ed.) *Medicine and Hygiene: Some New Perspectives* (衛生與醫療) (Taipei Shi: Academia Sinica, 2013).

92 For example, see Chiang, *op. cit.* (note 10).

93 Cai Yongji, ‘Introduction of Publication about Neurasthenia’, *Sin Chew Daily*, 19 August 1973.

94 Cunren Chen*, op. cit.* (note 47), 1140.

95

Shen-Kuan Yee et al., ‘Regulatory Control of Chinese Proprietary Medicines in Singapore’, Healthy Policy 71 (2005), 133–4910.1016/j.healthpol.2003.09.01315607377; Yan Yang , ‘A Brief History of Chinese Medicine in Singapore’, in Vivienne Lo , Michael Stanley-Baker and Dolly Yang (eds.) Routledge Handbook of Chinese Medicine (Milton Park, Abingdon, Oxon: Routledge, 2022), 524–36.

## Appendix

**Table A1.** Table of proprietary medicine listed on sale manuals in 1960, 1966 and 1970s^96^

No.	Product	Company	Price (HKD)	Unit	Targeted symptoms
1	Six-Ingredient Rehmannia Pill (六味地黃丸)	Ding Lou (鼎爐)	4.5	One box	Neurasthenia, headache, night sweat, deficiency of yin in kidney
2	Royal Jelly (蜂王漿)	Lai Ji Paai (荔枝牌)	8	30 tablets	Neurasthenia, fatigue, replenish health after illness or pregnancy, lack of appetite
3	Yeung Sam Ning San Yun (養心寧神丸) 1966	Yeung Sing Paai (羊城牌)	4.5	10 tablets	Neurasthenia, deafness, vexation
4	Lu Mei Ba Yun (鹿尾巴丸)	Yeung Sing Paai (羊城牌)	3	100	Neurasthenia, weakening heart, insufficiency of blood
5	Sam Yung Yin Ling Pin (參茸延齡片)	Lam Hoi Paai (林海牌)	4	100	Neurasthenia, kidney deficiency, vexation
6	Gwai Ling Jaap Yun (龜齡集丸)	Yun Ji Paai (遠字牌)	5	36	Neurasthenia, seminal emission
7	Bou Nou Yun (補腦丸)	Cheung On Paai (長安牌)	5	300	Neurasthenia, poor memory, fatigue,
8	Phosrose Tonic (艾羅補腦汁)	Reat Eastern Dispensary (Taiwan) Hong Kong Factory (中法藥房港廠)	/	1 doz size	Brain-fog, dizziness and unsound sleeplessness
9	Ling Sun Pills (寧神丸)	Wai Yuen Tong (位元堂)	/	6 pills per box	Neurasthenia and insomnia
10	Sunlight Pill (日光丸)	Leung Kai Fook Medical Store (梁介福藥行)	/	3 bottles	All kinds of depletion related to men, neurasthenia, weakening brain
11	Ning San Yun (寧神丸)	Wong Yiu Nam Medicine Factory (黃耀南製藥廠)	/	6 pills per box	Neurasthenia, poor memory, nightmare

